# The Epidemiological Scale of Alzheimer’s Disease

**DOI:** 10.14740/jocmr2106w

**Published:** 2015-07-24

**Authors:** Gavril Cornutiu

**Affiliations:** Clinic of Psychiatry, Faculty of Medicine and Pharmacy, University of Oradea, 26 Louis Pasteur Street, 410154 Oradea, Bihor, Romania. Email: g_cornutiu@yahoo.com

**Keywords:** Alzheimer’s disease, Epidemiology, Evolutionary trends, Risk factors control, Protective factors control

## Abstract

Alzheimer’s disease (AD) has increased from a few cases in a country at the beginning of the 20th century to an incidence of recording a case every 7 seconds in the world. From a rare disease it has reached the top 8 of major health problems in the world. One of the epidemiological problems of AD is the fact that authors from different countries use different reporting units. Some report numbers to 100,000 inhabitants, others to 1,000 inhabitants and others report the total number of cases in a country. Standardization of these reports is strictly necessary. The rise in incidence and prevalence with age is known, but interesting to see is that the incidence and prevalence do not rise in a parallel manner with age as simple logic would assume. Between the ages of 60 and 90, the incidence in men increases two times and in women 41 times, prevalence increase in men is 55.25-fold and in women 77-fold. Regarding the women/men ratio, the incidence is 20.5-fold increased, and prevalence is merely 1.3936-fold increased. These numbers raise concerns about the evolution of the disease. Regarding mild cognitive impairment (MCI)/AD ratio, only about 1 in 2 people get AD (raising?) issues about the pathogenic disease relatedness.

## Introduction

In order to talk about something specifically, we must first define that something [1]. It is generally agreed that we currently have three definitions.

### ICD-10 definition

At F00 code there are four subtypes of dementia in Alzheimer’s disease (AD): early onset Alzheimer dementia (< 65), Alzheimer dementia with delayed onset (> 65), Alzheimer dementia with atypical or delayed form as well as a subtype of Alzheimer dementia without any specification. The last two subtypes suggest a certain degree of relativity in the approach of AD, with a demarking area bordering other dementia pathological states, or even intersecting them; this may influence the incidence and the prevalence of the disease. As we can observe, ICD-10 distinguishes between dementia and the non-specific general dementia syndrome, even if not identically in all approximately 77 neurological diseases in which the dementia syndrome and the actual AD occur [2].

This is defined as “primitive cerebral degenerative disease with unknown etiology and with characteristic neuropathology and neurochemistry”.

It is obvious that the dementia syndrome, by its complexity and by the engagement of all psychic functions in the functional psychic failure, has the dimensions of a well-established disease, it is still the consequence of a neurological condition. If we consider dementia a disease, a pathological entity, then it can only be a disease following another disease. The wholeness of the human being obliges us to look at both sides of the coin. Theoretically, taxonomically and heuristically, the problem is open to discussions.

DSM-V^TM^ (2013) [3] replaces the term of dementia with the one of neurocognitive disorders (NCDs), a larger hat in which we also have: dementia syndrome, delirium, amnesic syndrome and other cognitive disorders, which, medically speaking (cause, symptomatology, evolution, treatment and prognostic), do not have much in common. This NCD from DSM has a double subdivision. The first subdivision is according to the intensity of the dementia symptomatology, in major and mild NCD, but the separation criteria of where mild NCD ends and where major NCD begins are adjectival and not quantitative, which again generates a degree of diagnosis relativity, influencing the epidemiologic accuracy. The second subdivision is probable and possible; the first one is conditioned by the presence of a family evidence of the disease and/or the genetic markers of the disease, and the second one by their absence.

Sporadic cases [4] come up to 25% of the total cases of AD with a probable diagnosis and hence we are getting close to what we might call pragmatic seriousness. This is due to the complexity of the pathology and to the absence of essential information at this stage of knowledge about the disease, because, as Osler [5] stated, “all scientific truth is conditioned by the state of knowledge at the time of its announcement”.

The third definition and the most complex one comes from the National Institute of Neurological and Communicative Diseases and Stroke/Alzheimer’s Disease and Related Disorders Association (NINCDS/ADRDA). This definition [6] mentions not just the clinical presence of the cognitive symptoms of the dementia syndrome confirmed by psychological and the neuropsychological tests but also the indirect histopathological criteria through typical imagistic examination. What NINCDS/ADRDA requested as “definite AD” diagnosis stage through histopathological examination, tends to be replaced today by neurochemical tests, but we have not yet reached a stable certainty of the value of their sensibility and sensitivity [7, 8].

With all the limitations of the diagnosis rigor, the three definitions of dementia in AD, the three sets of criteria, correspond in essence to the princeps case described by Alois Alzheimer. Even if there is a certain relativity of the limitations of the diagnosis definition, the epidemiologic data in specialized literature tend to agree on the issue of estimating the incidence and the prevalence of the disease but especially on the general evolutionary and local geographical tendencies of the morbidity evolution. According to general definition in statistics [9], incidence means “the number of new cases of given condition occurring within a specific time period” and by prevalence “the proportion of individuals with a condition within a specific population at a given time (point prevalence) or over a given time period (period prevalence)”.

Things are clear in terms of prevalence as the cases are diagnosed and recorded. As for incidence, we have a problem because a “new case” requires a “disease onset”. But what if a degenerative disease occurs? When is the clinical onset? This is always insidious, unnoticed, for both the patient and the ones around him. In addition, there are tolerant populations who only notice a pathological case when functional failure has installed. In this case there is only one solution: to consider a new case not from the moment of onset but from the moment of the official diagnosis. This is the moment in which, regardless of geography or culture, there was need for medical assistance. Actually this fact is valid for all degenerative diseases, as well as other diseases. We pointed out all these minor aspects of epidemiologic methodology in order to prove that in the case of momentary AD prevalence and incidence we can only ask for relative rigor. But this does not lead to long-term errors especially on the evolutionary tendencies of the disease; pragmatically, it cannot influence the understanding of the evolution of morbidity and the conception of its prophylaxis.

Yet we have a language problem, as there is a lack of consistency in the expression of the rate of the disease. There are authors who speak of number of AD cases reported in a hundred, a thousand, a hundred thousand people, and lately more and more reports mention the total number of AD cases in a given population, in US, in Europe, worldwide. But doctors in general have less practice with numbers and the different expressions of data in different articles confuse readers. There is need for a standardized expression of AD rate. There is need for associations and institutions specifically dedicated to AD and dementia in general to intervene at the WHO and to regulate these inaccuracies. In the text we will keep the authenticity of each writer.

## The Level of Neuropsycho Knowledge at the Time of AD Discovery

It was the time [10] of Oscar Wilde, George Bernard Shaw, Henry James, Roentgen, who, in 1905, laid the foundation for radiology, the Lumiere brothers started cinematography, the first photoelectric cell was created, the first Diesel engine was built, the notion of geochronology was used, Becquerel discovered radioactivity, the first motorcycles showed, aspirin was invented, etc. Those were times of high intellectual effervescence. As for neurosciences, in the English speaking world, Jackson and Maudsley made great contributions. In 1892 a Romanian, Marinescu, and a French, Blocg, first described the senile plate of amyolid, found postmortem at the epileptic chronicle writers [11, 12]. These plates will turn into the current amyolidic syndrome which occurs in several degenerative diseases, especially in dementias. But the critical mass of psychoneuro sciences and of psychiatry in particular, is reached in Germany where Kahlbaum and Wundt influenced Kraeplin [13], Alzheimer and Nissl and where the preoccupation for pathology of the elderly was engaging more and more scientists. In Munich, Kraepelin, Alzheimer and Nissl formed a team. In Prague, Pick describes the disease which bears his name. If the Greeks discovered the thinking method, moving the antique world forward at a fast pace, the Europeans of mid-19th century discover the research method, revolutionizing the world back then. In this ideal atmosphere, Alzheimer notices the pathological atypical princeps case with onset at the age of 51 and with an evolution of 4 years and a half and publishes his observations in 1906 - 1907. In presenting the case, Alzheimer writes “in the past years, such particular affections have been recorded in large numbers, which stimulates us to study and analyze this condition in the future” [14]. The cases were so “numerous” only 4 years later, in 1910 Kraepelin, who names the disease, only had the description of six cases from two countries: Germany and Italy.

## The Current Rate of AD

From six cases discovered by three researchers in 3 years in two countries, we are now experiencing a new AD case recorded at every 7 s worldwide [15]. For USA, the Alzheimer Association (2013) reports a case every 6.8 s [16]. Considering these numbers and comparing the population of US with the world population, we can draw a quick conclusion that there are big if not huge differences in the real prevalence of the AD cases, in their rigorous diagnosis and in their reporting worldwide. Therefore, the global data we have must be regarded with flexibility and through what Kant [17] defined as the disjunction between the “actual reality and the perception of the given reality”. The data are not false, they represent a reality, but they must not be approached rigidly. Given the difference of epidemiological rigor throughout the world, we can be more than sure that the amplitude of the phenomenon is only partially known. It is certain that during the 108 years since the disease was named, the disease grew from six isolated cases into a truly serious health problem worldwide, ranking among the first eight major health problems of humanity. Apparently, the first half of the 20th century did not generate any stress among specialists or health organizations. In a study dating from 1963 [18], Vernaardt is quoted, who in 1950, while doing epidemiological studies, found the Pick disease in Indonesia, but no AD. Whereas in 2000, Indonesia reported 1,000,000 cases of AD to the WHO [19].

Other authors [20] of the same historical period (1959) compared the same country with two different geographical areas (Goetheborg and Stockholm) and found that AD was present in the first area and Pick disease in the second. In the 6 - 7 decades of the 20th century, the “natural occurrence” of AD was - in terms of prevalence - much higher than the one of Pick disease. Actually, in 1976, Predescu [18], wrote according to the literature back then “AD is more frequent than Pick disease”, so the difference was hardly significant. What is the situation today? We are going to give guiding answers.

For Pick’s disease we have [19] a rate of 7 - 43/100,000 inhabitants in 2007 in Europe and a rate of 28/100,000 inhabitants for 2008 in Holland [21, 22]. For AD we have in Europe [23] a rate of 504,000 cases (360,000 - 626,000). The first thing to notice is the heavy method of comparison for such data. Second of all, if we take into account that the Pick disease was described in 1892 and the pathological characteristics were defined only in 1911 (after 19 years!) by Alzheimer, we can deduce that was just as rare as AD at the turn of the 20th century and today it is expressed in figures which are far from small, but still its incidence and prevalence has grown but not at the same pace as the incidence and prevalence of AD. Both diseases are degenerative. For both of them the phenotypical expression of the genetic vulnerability like with all diseases is shaped by the environment [24]. The difference is in the impact of environmental factors on the incidence of AD compared to Pick’s disease. For AD the impact was explosive and the tendency stays explosive. This means that the mechanisms of the disease are innate by accumulation of vulnerabilities, but the environment can increase these vulnerabilities and start the mechanisms of the disease. This leads to the huge current prevalence of the disease compared to the initial moment when it was first described and has an explanation in the alteration of the environment in which human kind exists.

After the huge increase over the past 100 years, what we know very well is that AD prevalence increases fast with age. AD prevalence doubles every 5 years after the age of 60 [25, 26]. The rate ratio of > 85 years of age and that of 65 - 69 years of age is of 20.33 [27]. In a local study we found the following curve, whose amplitude varies temporally [28].

**Figure 1:**
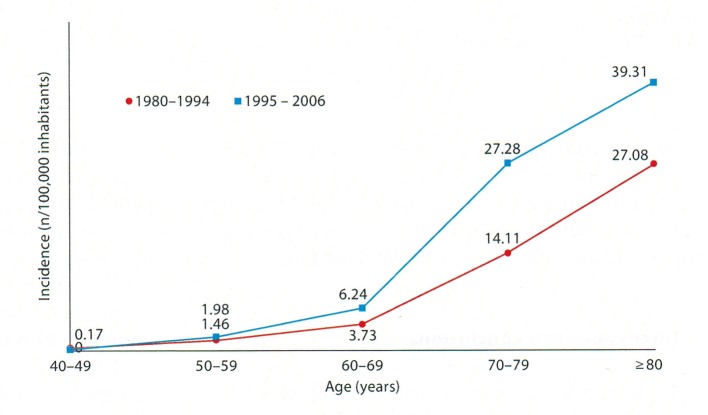
The variation of AD incidence in age groups during two periods of time [28].

In this study regarding the prevalence on two time segments and on age groups (Fig. 1), we notice that the first period 1980 - 1994, the ratio of the prevalence at the age of 80 and at the age of 50 - 59 is of 18.54 and for the period 1994 - 2006 the same ratio is of 19.85. In both cases, the curve rises sharply after the age of 60 but amplitude of the growing prevalence is not time consistent. The relation of AD prevalence with the age groups is unanimously accepted [29]. There is a classical appreciation made by Doodys [30] according to which in USA, AD had a prevalence of 12% for > 65 years old, 23% for > 75 years old and 47% for > 85 years old in 2010. Even if these numbers cannot be found in other geographical areas and in another time segment, they have big suggestive and guiding power, without taking away too much of the rigor science imposes, but which is at the same time dynamic. In fact, the static numbers pointed out, from the temporal point of view, they give the estimation of a moment, the phenomenon is best described by evolution curves, and it is the ratio between photography and film.

Owing to its strong connection with age as a pathological variant of ageing, the global data are according to the dynamics of population ageing process [31]. Cummings et al [32], starting from the research found by Jorm in 1991 [33] which found AD in 1% of the 60 years old population, 2% for 65 - 70 years old, 4% for 71 - 74 years old, 8% for 75 - 79 years old, 16% for 80 - 84 years old and 30-40% of the population over the age of 85 and from the population aging tendency, appreciated that the total number of AD case will increase from 1997 to 2025 as follows: in Africa from 2 million to 5.4, in Americas from 13.8 to 29.8 million, in Europe from 23.3 to 42.6 million, in south-east Asia from 6.5 to 24.3 million and in West Pacific zone from 16.5 to 51.8 million. According to epidemiologic data from Harvard [34, 35], in USA there were between 4 and 5 million AD cases in 2013.

According to Bachmann et al [36], “the incidence of dementias and probably of AD” increases from 7/1,000 inhabitants for 65 years of age to 118/1,000 inhabitants for > 85 years of age, which is a 16.65 times increase. According to McCuster Alzheimer’s Research Foundation in Australia [35], there were 245,000 cases of AD (> 1% of the population) in 2009 while there were only 52,000 cases in 2009. In 4 years, then, the incidence of diagnosis increased 4.71 times. This represents a real boom.

For India, Chandra and collaborators [37] found an incidence of 1.74/1,000 for the age of ≥ 55 and 4.7/1,000 at patients > 65. For India and other geographical areas such as Africa, where there is high birth rate and the age group ratio is still ascending for childhood and adolescence, the rate of the disease is still low. There is a reverse ratio between ageing and birth rate, which is valid for the relation AD incidence and birth rate. But when birth rate due to its effects will decrease, AD will boom in these areas, too. The tough question is whether in the meantime the medical assistance possibilities for AD patients will improve in these parts of the world.

In Denmark, Andersen and collaborators [38] found a rate of 20.9/1,000 inhabitants, without a difference between M/W. Unlike the Danes, the Alzheimer Society in Canada found that 72% of the AD patients are women, whereas men prevail in other dementias. They explain that the higher percentage of women patients is due to their longer life span and the difference between average life span for the two gender groups. Also, they estimate that the number of AD case will double in the next 25 years [39].

In Japan [40, 41] AD increased from 1.1% in 1985 to 3.8% in 1992. Brookmeyer and collaborators in 2007 [42] appreciate that there were 26.6 million cases of AD in the world in 2006 and in 2050 one person in 85 will suffer form AD (1.176% of total population) or 106.8 million, with limits between 47.2 and 221.2, will suffer form AD and that 9.2 million AD patients will require institutional care, be it in specialized units or at home. They foresee an annual incidence for ≥ 80 years of age of 1.48% yearly, with limits between 0.67% and 3.4%. They also deny the geographical differences, considering that the differences occur due to different diagnostic processes. But if the phenotypical expressions of AD vulnerabilities depend of environmental factors [43], fact that is unanimously accepted, then we must admit that environmental factors, socio-demographic factors, food, etc. differ from on geographical area to another. This fact makes Brookmeyer’s and his collaborators’ statement a mere arrogance. Actually, Hendrie and collaborators [44], a joined team made up of specialists from Indianapolis (USA) and Ibadan (Nigeria), making an assessment based on unique criteria by the same people of AD prevalence in Afro-Americans from Indianapolis (80% of population) and Yoruba population (prevailing) in Ibadan, found an AD rate of 1.15% (with limits between 0.96% and 1.35%) in Ibadan and 2.52% (limits between 1.40% and 3.64%) in Indianapolis. There are no genetic studies to this date to indicate that there are populations with more genetic vulnerabilities for AD than others. There is the Rotterdam study in 1995 to support the conclusions of Brookmeyer team conclusions that the geographical differences are due to diagnostic differences. The team found that AD was sub-diagnosed, confirming the association of prevalence with age.

Actually, Ott and collaborators in 1995 [45] draw the conclusion that prevalence could be higher than previously accepted. But none of the studies discusses the rapid growth of incidence from one decade to another and studies of prevalence for the one and the same place are rarely conducted in a repetitive manner, so that theoretically, the differences can be found in three sources: geographical differences, incidence increase and diagnostic differences. Brookmeyer and collaborators in 1989 [25] found a number of 2.32 million AD patients (limits between 1.09 and 4.58 million), assessing that in 2050 one in 45 people will suffer from AD (2.2%). For the same geographical area, Evans in 2003 [46] asserts that if in 1980 there were 2.88 million people suffering from AD at the age of ≥ 65 years, in 2050 there will be 10.3 million such cases.

As we can see, studies from the same country can hardly be compared. At global level, the Delphi study in 1994 [47] stated that there were 24.3 million AD cases that year and that the cohort rises yearly with 4.6 million cases, a new case for each 7 s and that there will be 81.1 million AD cases in 2040. The same study asserts that the growth of prevalence rate will not be uniform. If the cohort will rise 100% for developed countries between 2001 and 2040, for China, India and south-east Asia, it will increase 300%. Purohit and collaborators in 2011 [48], after analyzing the Indian specialized literature, point out the prevalence dynamics, which depends on the dynamics of growing ageing population, and which, in its turn depends on the general demographic evolution. According to Hebert and collaborators [49], there were 4 - 5 million AD cases in USA in 2002 and there will be 13.2 million cases in 2050, a threefold increase. But, comparing the epidemiological data concerning the situation in the USA, we notice a pitiful shallowness of figure estimations. The conclusion is the same: the lack of a generally accepted standardized methodology, a universal tool which would make the results obtained through research compatible.

Mura and collaborators in 2010 [23] estimate for France a number of 754,000 people with dementia in general, 1.2% of the population, which would increase to 1,813,000 in 2050, 2.6% of the population. The same team [23] estimated for Europe a number of 7.21 million dementia cases in 2006 and for 2050, a number of 16.51 million cases (the eighth nation in Europe!). For AD in France, they estimate a number of 504 cases/1,000 inhabitants for 2010, with limits between 360 and 626. In Europe there were 6 million people with AD in 2010, 74.3% women. In 2050 in Europe there will be 14.5 million cases. This means that in 2010 the prevalence was of 754/1,000 people, with limits between 614 and 958 and in 2050 there will be 1,813/1,000 people with limits between 1,428 and 2,373. For Spain 2009, Ortega and collaborators [50] found a prevalence of 10.9% with important regional variations between 3.5% in Bidasoa and 17.2% in Pamplona.

Reitz and collaborators [51] estimate the following rates for 1,000 inhabitants: 10.5 for North America, 8.8 for Europe, 9.2 for Latin America, and 8 for China and the Pacific Zone.

Center for Disease Control and Prevention [52] estimates that in 2013 in USA there are 5.3 million cases of AD. They also show that AD is the sixth cause of death in the USA and the fifth for people > 65. Mayeux and Stern in 2012 [27] appreciate that globally there are 24 million people suffering from AD with a cohort which doubles every 20 years. The Alzheimer’s Association report for 2012 asserts that there are one million new cases of AD every year worldwide [53-55]. WHO estimated that in 2012 there were approximately 7.7 million new cases of AD worldwide, a new case every 4 s, and the new cases < 65 stand for 2-10%. Alzheimer Europe [56] states in the Declaration of Paris that in 2006 there were 5.4 million Europeans with AD, with the probability of doubling until 2040.

### The incidence-prevalence ratio

The most complete report is the one in EURODEM study, a meta-analysis of 11 studies from eight European countries. Table 1 shows the EURODEM analysis on age groups form 60 - 64 years of age to ≥ 90 and on sexes [56].

**Table 1:** The Dynamics of Incidence and Prevalence Related to Age, According to the EURODEM Study

Parameter	Incidence rate		Prevalence rate	
	Men (M)	Women (W)	Men	Women
60 - 64 years	0.2	0.2	0.4	0.4
≥ 90 years	0.4	8.2	22.1	30.8
Increase ratio	2 times	41 times	55.25 times	77 times
Final ratio: women/men	about 20.5		1.3936	

The evolution of AD incidence and prevalence percentage is interesting to watch. So, for the most inferior age group of 60 - 64 years, the incidence is 0.2 and prevalence from 0.4. From here on, every incidence and prevalence on sexes grows progressively to reach ≥ 90 at the following figures: 1) for M incidence increases from 0.2 to 4; 2) for W it increases from 0.2 to 8.2; 3) for M prevalence increases from 0.2 to 22.1; 4) for W prevalence increases from 0.4 to 30.8. Therefore, we can express growth in multiples between the two age groups.

Therefore, the incidence in M grows two times; the incidence in W grew 41 times; prevalence in M increased 55.25 times; prevalence in W increased 77 times. The increase is greater for W both for incidence and prevalence. But there is a ratio difference between incidence and prevalence in the respect of the decreasing ratio for prevalence in M/W. This means that women live shorter lives after the onset of the disease. These differences should be systematically checked because if they are real, they pose pathogenic problems.

Temporally, the Alzheimer association estimates that in the USA the global incidence will increase between 2012 and 2050 from one case every 6.8 s to one case every 3.3 s, which represents a twofold increase. For prevalence in the same area and time, they estimated an increase from 11 milion to 16 million cases of AD, meaning a 1.45-fold increase. Brookmeyer and Gray in 2000 [57] estimated that in the next 50 years, the incidence will increase four times, therefore incidence does not relate to age groups just linearly but other favoring factors for incidence increase intervene.

Both incidence and prevalence were evaluated on a limited number of people and a global estimation on states, regions or worldwide depends on the applied mathematical models. That is why the estimations have a limitation in terms of accuracy and must be regarded as reference terms not as absolute realities [58]. The value of incidence estimation and of prevalence should enable the finding of some risk factors [38].

## Medium Cognitive Impairment and Its Relation With AD Prevalence and Incidence

It can be triggered by all pathogenic states which affect the structure and the function of specific neuronal population. Mild cognitive impairment (MCI) is, therefore, a non-specific syndrome. But as Hughes and collaborators stated in 2011 [59] that the delimitation of some subtypes cannot be done even if most people over the age of 80 have MCI, and approximately half of them end up with dementia, as Lopez and collaborators conclude [60] after studying 285 cases. In this study the incidence of MCI was of 111.09 in 1,000 people. Out of these, 53.5% experience a form of dementia in an average time span from MCI diagnosis, varying from 2.8 ± 1.8 years.

But a report of Mayo Clinic [61] points out that MCI may increase the risk of developing a dementia in AD “but some people with MCI never get worse, and a few eventually get better”. The researchers’ stake in terms of MCI is high because, just like in the case AD, the same therapeutic principle applies: “the sooner applied, the more efficient”. Ganguli and collaborators [62] conclude in 2011that “MCI is a heterogeneous entity at the population level but progresses at rates higher than in normal elderly individuals. Proportions of participants progressing to dementia are lower and proportions reverting to normal are higher than in clinical populations”. In the report for 2011 - 2012 NIH [63] confirmed and used Ganguli and collaborators’ study as reason, stating that “They found that only a small proportion of those who met the diagnostic criteria for MCI progressed to dementia over the course of 1 year; the majority remained stable, and a few even improved”.

In another study dedicated to the relation between MCI and the vascular risk, in 2013, Ganguli [64] and his team find an incidence of 95, respectively 55 in 1,000 inhabitants by using “neuropsychological (NP) - MCI and functional (clinical dementia rating (CDR) = 0.5) for the definition of MCI. They also find that MCI incidence does not vary with sex or education but it varies according to age. Andersen and collaborators in 1996 [38] after following a cohort of 2,452 people on two age groups: 65 - 69 years of age and 80 - 84 years of age for 2.1 years, found that the incidence for “very mild dementia” in the 65 - 69 groups was of 8.6 in 1,000 people and it increased to 20.6 for the 80 - 84 group with a global average of 15.3. For “very mild to severe dementia” they found an incidence of 12.4 in 1,000 people for the 65 - 69 age group and 82.2 for the 80 - 84 years age group, with global average of 29.5. Therefore, comparing the 65 - 69 and 80 - 84 age groups, we notice a threefold increase for very mild cognition and a 6.6-fold increase for very mild to severe dementia.

If we corroborate these numbers with AD incidence we have the surprise to notice that there is a positive concentration of cognition affection with ageing. This is probably due to the fact that demographically, there are less and less people over the age 65 around. We reach then the unpleasant conclusion that dementias are the price we pay for living longer in the current living style and environment. But both living style and environment represent relatively controllable parameters, which would allow for a positive perspective yet rationally limited.

## Favoring and Protective Factors for AD Incidence and Prevalence

One must differentiate between the demographic AD phenomenon and the individual AD phenomenon. The demographic phenomenon implies the epidemiological amplitude of the disease and the factors which influence the evolution of this amplitude. The individual AD phenomenon implies the trigger mechanisms, the AD maintenance factors as well as the individual vulnerability record of the clinically manifested or not manifested case. The articulation of the two phenomena is given by the factors which influence the epidemiologic amplitude of the disease and the person’s vulnerability record. Therefore these risk factors and the protective factors are equally important from the individual or epidemiological point of view.

### Risk factors

#### Age

The first risk factor is related to age. The older the person, the more likely he is to develop AD, as all epidemiologists pointed out. The second risk factor is heredity [27, 65, 66]. The vulnerabilities detected to the present day refer to mutations in chromosomes 21 and 14 [67, 68]. Transmission is dominantly autosomal with a complete penetration which is expressed phenotypically quite early, before the age of 65 but which represents less than 5% of the total cases [32]. These are uncontrollable risk factors currently and probably definitively, but we also have controllable risk factors, such as: minor vascular accidents, HAT, diabetes mellitus (DM), smoking, depressions, body weight, cerebral commotions, high plasmatic level of lipids, insomnias, depressions, repeated stress, behavior of certain personality profiles, eating habits, informational isolation, sedentary life and the inaction of the elderly.

#### Strokes

Pendleburi and Rothwel [69] conclude as a result of a meta-analysis that over 7% of patients with a first stroke develop dementia later on. Wen and collaborators in 2007 proved that [70] minor transitory ischemic strokes lead to production of tau protein. Jellinger in 2002 [71] proves that transitory ischemic strokes affect the paranchimose structure of the brain, and those with strategic location, like thalamic geography, give memory loss.

#### Arterial hypertension

Kilander et al in 2000 [72] and Whitmer et al in 2005 [73] among other authors claim, as a result of clinical demonstration, that middle aged people who had hyper blood pressure developed dementia more frequently than the general population. Yoshitake et al in 1995 [40] and Sekita et al [41] after studying 828 patients observed that age, high blood pressure and alcohol are risk factors for vascular dementia (VD). For AD age was advanced. In the meantime Sekita and collab notice that for the past two decades, the prevalence of AD and the prevalence of VD increased simultaneously in Japan. Knowing that VDs Alzheimer and that AD dementias gain vascular features in time, we are faced with a methodological problem: from which point do we talk about a mixed vascular and Alzheimer dementia dominantly Alzheimer and from which point do we talk about a dominantly vascular one? Or is there a point in making a disjunction? The final point of all demential syndromes, regardless of the disease which produced them is a vegetative state with a cvasi-absent psychism. At which point do we lose the specificity of a demential syndrome related to a disease?

#### DM

Most authors [74-77] assert that the relation AD-DM is specific for average cases. DM does not induce MCI or AD but in comorbid cases MCI + DM increases with 20-70% the conversion of MCI in AD compared to the cases of MCI without DM.

#### Nutrition

In 2011, Cornutiu [28] published the conclusions of a study on the evolution of incidence and prevalence of AD in the population of a county in Romania. Until 1989 the Romanian population lived in isolation, with secured borders. Nourishment was scarce, but ecological. After 1989 the borders were open, commerce was free and the food market was flooded with non-ecological but appetizing products for people used to the gray and dullness of the communist bloc. The results were as follows: between 1980 and 1993 the incidence varied around the value of 1.65 cases in 100,000 people, with insignificant yearly changes. Three years after the introduction of non-ecological food, the incidence of AD rose sharply and in 2006 it reached an average of 7.05, with a 3 years’ average (1994 - 2006) of 3.43, more than double. We observed all parameters in the study: medical, social, demographic, age, psychological and alienation. None of them had changed significantly over these years. The only major change was the replacement of ecological food with the appetizing non-ecological one for an unwarned population. The conclusion is obvious: nutrition can generate the rise or boom of AD incidence or prevalence or it can diminish it. Grant in 1997 [78] publishes a study in which he shows that in the USA, African Americans and Japanese people have a higher prevalence of AD than in their original countries, due to different customs and eating habits. More importantly, the author finds 11 countries with a significant correlation between AD prevalence and extra fat and calorie intake. On the other hand, nutrition based on fish, vegetables and fruit diminishes AD incidence.

Obviously, there is a close parallel between the progressive rise in the AD incidence and prevalence and global industrialization of human nutrition, with almost all food coming from chemically cultivated plants, artificially reared animals, processed and preserved food, etc. This is the only parameter that has changes just as much as AD incidence. Life expectancy rose mildly, compared with AD incidence rise.

In Japan, for instance, the country where there was the highest proportion of people over the age of 100, the sharp increase in the AD incidence occurred parallel to westernizing the country and changing eating habits. Currently we have evidence of the relation between AD incidence and a decrease of vitamin D3 in the blood [79, 80].

Fratiglioni [81] stated in 1996: “Among the factors that have been investigated, only age, familial aggregation, and apo-lipoprotein E gene-e4 allele are definite risk factors both for early and late onset AD. However, many of the possible and putative risk factors, if definitively confirmed, can be prevented or controlled”. Indeed, apart from innate factors and age risks, the rest of the factors are controllable, and the most important one, the nutritional one, requires a more consistent approach, better knowledge of available products and eating habits as well as the establishment of a social-political-medical monitorization as soon as possible.

### Protective factors

Among the protective factors, many authors point out the nutritional factor (diet) [82-87]. They all point out the protective effect of ecological and rational food. Barberger-Gateau and collaborators in 2013 [88] point out role of Omega3 acids in fish and fruit, with high level of antioxidants in diminishing the phenotypical expression of AD type vulnerabilities. They specifically draw attention to the low level of AD prevalence in the Mediterranean areas.

Therefore we have the demonstration of effect of AD incidence rise, of non-ecological food as well as the protective effect of ecological food. The protective role of cognitive training, of continuous mental activity was also pointed out by [37, 89-92]. Maintaining a muscle tone by doing daily activities, as an anti-dementia protective factor was pointed out among others [93-96]. To sum up, protective factors refer to a natural life, in the manner and conditions in which man has evolved and adapted over generations and the risk factors are represented by parting with such life style. All studies of protective and risk factors for AD point out the utmost importance of rational use of all remaining functional capacities of the elderly. Standard retirement is a risk factor for AD incidence.

## Conclusions

Globally, the data show that AD incidence and prevalence have been on the sharp rise for past 50 years and that AD has evolved from a rare disease into the eighth greatest health issue worldwide. This evolution happened simultaneously to the man’s desertion of the ecosystems to which they have adapted throughout existence, simultaneously to the alteration of human existential condition, especially nourishment. The risk factors influencing the level of AD incidence and prevalence are of two kinds. We first have the uncontrollable factors which are innate and come with age, with a rising life expectancy. But these are minor factors compared to the controllable factors which we are getting acquainted to and which we should impose on the collective mind and on social and political decision factors.

Therefore, the AD epidemiological analysis imposes the following measures as an emergency for the control of AD rate: 1) a standardized expression of the rate of AD demographical phenomenon, both in scientific papers and in official reports; 2) socio-medical supervision of controllable AD risk factors, especially food related (with precise production, processing and preserving norms), with the involvement of political and social decision factors; 3) socio-economical analysis of the non-standardized retirement opportunities, more exactly, promoting individualized retirement on medical criteria of remaining functional potential, in order to preserve the population health state.
